# Exon-Specific QTLs Skew the Inferred Distribution of Expression QTLs Detected Using Gene Expression Array Data

**DOI:** 10.1371/journal.pone.0030629

**Published:** 2012-02-16

**Authors:** Jean-Baptiste Veyrieras, Daniel J. Gaffney, Joseph K. Pickrell, Yoav Gilad, Matthew Stephens, Jonathan K. Pritchard

**Affiliations:** 1 Department of Human Genetics, The University of Chicago, Chicago, Illinois, United States of America; 2 BioMiningLabs, Lyon, France; 3 Howard Hughes Medical Institute, Chevy Chase, Maryland, United States of America; 4 Department of Statistics, The University of Chicago, Chicago, Illinois, United States of America; University of Sheffield, United Kingdom

## Abstract

Mapping of expression quantitative trait loci (eQTLs) is an important technique for studying how genetic variation affects gene regulation in natural populations. In a previous study using Illumina expression data from human lymphoblastoid cell lines, we reported that cis-eQTLs are especially enriched around transcription start sites (TSSs) and immediately upstream of transcription end sites (TESs). In this paper, we revisit the distribution of eQTLs using additional data from Affymetrix exon arrays and from RNA sequencing. We confirm that most eQTLs lie close to the target genes; that transcribed regions are generally enriched for eQTLs; that eQTLs are more abundant in exons than introns; and that the peak density of eQTLs occurs at the TSS. However, we find that the intriguing TES peak is greatly reduced or absent in the Affymetrix and RNA-seq data. Instead our data suggest that the TES peak observed in the Illumina data is mainly due to exon-specific QTLs that affect 3′ untranslated regions, where most of the Illumina probes are positioned. Nonetheless, we do observe an overall enrichment of eQTLs in exons versus introns in all three data sets, consistent with an important role for exonic sequences in gene regulation.

## Introduction

Polymorphisms that impact gene regulation play an important role in disease genetics and adaptive evolution [1], [2]. One important tool for identifying such variants is by genome-wide mapping of expression quantitative trait loci (eQTLs) [3]–[5]. A number of recent studies have aimed to characterize the properties of genetic variants that produce eQTLs, including the types and locations of variants, and the functional context of the variants in question [6]–[12].

In previous work, we developed a Bayesian hierarchical method for studying the distribution of eQTLs with respect to their target genes, and for identifying biological anotations that can predict the locations of causal sites [9], [11]. Applying that method to Illumina expression array data collected in the HapMap lymphoblastoid cell lines, we observed a striking enrichment of eQTLs immediately upstream of the TES [9], in addition to a more expected enrichment around the TSS of the target genes [7], [8]. In that paper we proposed two main hypotheses to explain the presence of the eQTL peak in the 3′ UTR: (1) these may be variants that affect stability or degradation of the entire transcript (e.g., miRNA binding sites), or (2) these may be variants that affect inclusion levels of the last exon (e.g., splicing QTLs; note that most of the Illumina probes lie in the last exon). In the original paper we argued that the first mechanism was likely to be most important. However, in a more recent study using RNA sequencing to measure isoform expression levels for a subset of the HapMap LCLs we did not find a peak of eQTLs at the TES [13].

Here we revisit the TES peak to understand better the mechanism that generates this intriguing signal. Using expression data for the same samples from independent experiments and different technologies, our new analysis suggests that in fact exon-specific effects are responsible for most, if not all of the 3′ UTR peak that we saw previously. However, we find that our previous result showing an eQTL enrichment in exons overall, compared to introns is supported by all three data sets.

## Results

### Datasets and cis-eQTL mapping

For this analysis we used data from the 210 unrelated HapMap samples in the original HapMap Phase I/II cell lines [14]. These include 60 CEPH (CEU), 60 Yoruba (YRI), 45 Chinese (CHB) and 45 Japanese (JPT) samples. The SNP genotypes were based on HapMap genotypes for all HapMap SNPs, combined with whole-genome sequence data from the 1000 Genomes Project [15]. For individuals not sequenced by the 1000 Genomes Project, missing SNP genotypes were imputed using BIMBAM [16], [17]. See the Methods for further details.

We analyzed expression measurements obtained using three distinct technologies:

- Illumina gene array data from a total of 210 CEU, CHB, JPT and YRI samples [6] and hereafter referred to as the Illumina data set. This is the expression dataset we used for our previous study [9];
- Affymetrix exon array data from 117 CEU and YRI samples [18] and hereafter referred to as the Affymetrix data set;
- RNA sequence data from 102 CEU and YRI samples [13], [19], hereafter referred to as the RNA-seq data set.

Note that the 117 individuals in the Affymetrix data set, and 102 individuals in the RNA-seq data set are both subsets of the 210 individuals in the Illumina data set (and in both cases include the majority of the CEU and YRI samples). The original RNA-seq data sets included a few individuals that were not in either the Illumina data or Phase I/II HapMap; in order to simplify the genotype imputation pipeline these individuals were excluded from the analysis.

To avoid the impact of spurious associations caused by SNPs falling within the probes of the two array data sets (Illumina and Affymetrix), we systematically removed all probes containing at least one SNP. We also removed all probes impacted by short insertions/deletions or copy number variations (CNV) based on the genomic coordinates of these structural variants as provided by [20] and the 1000 Genomes Consortium [15]. Finally, for all expression data we removed probes (Illumina and Affymetrix) or exons (RNA-seq) that appear to be “non-expressed” (see Materials and Methods). For each dataset, expression levels were quantile normalized to a standard normal distribution within populations prior to performing the mapping of cis-eQTLs, in order to avoid false positives due to population structure [9]. More extensive details on the data processing are provided in the Materials and Methods section. Table S1 provides a summary of the content of each expression dataset.

For eQTL mapping, we used standard linear regression to test every SNP within the transcript or 100 kb from either end of the transcribed region for association with gene expression. Table S1 reports the number of eQTLs we found for each expression dataset for an empirically estimated False Discovery Rate (FDR) of 5%. For each gene with at least one significant SNP, we treated the most significant SNP as an estimate of the major eQTN (Quantitative Trait Nucleotide) for that gene. When more than one SNP had exactly the same lowest p-value (e.g., several SNPs in perfect LD) we equally shared the probability of being the major eQTN among all these SNPs.

### The TES peak is strongest in the Illumina data and absent in the RNA-seq data

**Figure 1:**
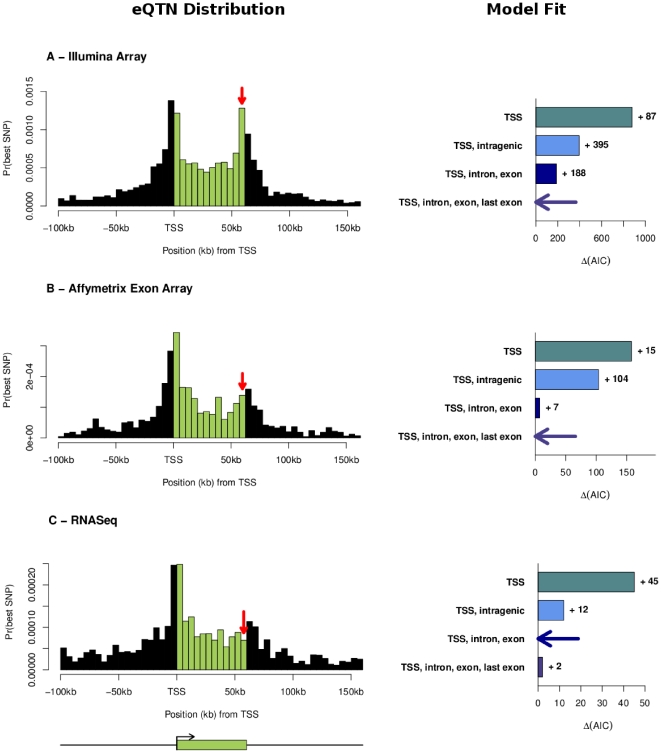
Expression QTN distributions estimated using three different technologies for measuring gene expression. The left-hand column plots the distribution of locations of most significant SNPs for each technology; the red arrows indicate the location of the TES peak observed in the Illumina data. SNPs outside genes are assigned to bins based on their physical distance from the TSS (for upstream SNPs), or TES (downstream SNPs). SNPs inside genes are assigned to bins based on their fractional location within the gene. The plotted gene size is the average gene length in the data. To provide a formal comparison among different models, the right-hand column displays the difference in Akaike Information Criterion (AIC) values between different parameterizations of our Bayesian hierarchical model (see Methods and Table 1). Small values of 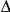
“(AIC”) indicate better model fit, and the best model for each data set is indicated with a horizontal arrow. The labels for the four models indicate the different parameters included in each model: “TSS” refers to our basic distance model measured as distance from TSS; “intragenic” means that we use a single additional parameter for all SNPs within the transcript; “exon, intron” indicates that we use separate parameters for exonic and intronic SNPs respectively, and “last exon” indicates that we add an additional parameter for SNPs in the final exon.

The left panels of Figure 1 display the distribution of locations of the best SNP with respect to the target gene (similar to Figure 2 of [9]): the vertical red arrow on each panel highlights the location of the Illumina TES peak. As is evident, the peak upstream of the TES is strongest in the Illumina data, weaker in the Affymetrix data, and essentially absent from the RNA-seq data.

**Figure 2:**
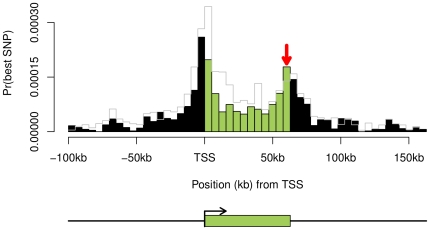
Expression QTN distribution estimated using only those Affymetrix probes that are located within the same exon as an Illumina probe creates an apparent 3′ signal peak. Overall, the Affymetrix probes are spread roughly evenly across exons while the Illumina probes are 3′ biased. By analyzing only those Affymetrix probes that are in the same exons as Illumina probes, we create an apparent 3′ signal peak. For the sake of comparison, the grey line represents the original distribution as plotted in Figure 1.

To assess the evidence for a TES peak more quantitatively, we computed the AIC (Akaike Information Criterion) for a model with, and without a special TES effect (Figure 1; compare the “TSS, intron, exon” model to the “TSS, intron, exon, last exon” model). As illustrated in the figure, the model with a special effect for the last exon (i.e, the exon ending at the TES) is strongly preferred for the Illumina data (by 188 units of likelihood), it is weakly preferred for the Affymetrix data (by 7 units), and weakly disfavored for the RNA-seq data (by 2 units).

One major difference between the Illumina data and the other two data sets is that a large fraction of the Illumina probes are positioned in the last exon (85% for Illumina compared to 21% for Affymetrix). To assess whether the Illumina probe placement might have helped to create the peak of signals at the TES, we filtered the Affymetrix data to include only those Affymetrix probes that are in the same exon as an Illumina probe (and hence the filtered data set includes mainly probes in the last exons of genes). When we did this, we observed that indeed the filtered Affymetrix data set showed a much stronger peak of eQTLs in the last exon (Figure 2). This latter observation strongly suggests that the probe distribution on the Illumina array has helped to create an apparent peak of eQTLs in the last exon that is not supported by the other data sets.

In principle, one plausible explanation might be that ungenotyped SNPs in Illumina probes could generate spurious eQTL signals, and that these would often be detected by nearby SNPs; such an effect might in principle generate a spurious 3′ peak of eQTLs. However, this does not appear to be the case. The original analysis of Veyrieras *et al* included a correction factor for unmeasured SNPs-in-probes that suggested that the effect of such SNPs was relatively modest [9]. That conclusion is confirmed by the analysis presented here, for which we removed all probes containing 1000 Genomes SNPs (which should include nearly all common SNPs in probes); the distribution of eQTNs in the Illumina data is very similar to the corrected distribution reported previously [9].

### eQTNs within last exons frequently have exon-specific effects on expression

Recent work has shown that there are many SNPs that impact the expression levels of individual exons, while not necessarily affecting the overall expression levels of genes [13], [19], [21], [22]. Such QTLs are often referred to as “splicing-QTLs” (sQTLs) although in some cases they arise through other mechanisms than splicing changes *per se* (for example, changing the transcription end site position [22]). Fraser and Xie, who also analyzed the Affymetrix data, reported that the last exon is particularly prone to exon-level QTLs [21]. Given these observations, we conjectured that the TES peak of eQTLs in the Illumina data may be caused by SNPs that lie in or near the last exon, and that affect expression levels of the last exon only.

To evaluate this hypothesis, we computed exon-specific expression levels in each individual using both the Affymetrix and the RNA-seq data sets, controlling for the overall expression level of the gene in that individual to remove the impact of gene-level eQTLs (see Materials and Methods). We then tested whether eQTNs identified using the Illumina data would replicate at either the exon level (as sQTLs) or at the gene level (as eQTLs) in the Affymetrix and RNA-seq data sets. For each gene with a significant eQTL in the Illumina data set (FDR = 5%), we designated the most significant SNP as a putative Illumina eQTN. (In many cases the putative eQTNs will not be the true causal sites, but they should at least be in strong LD with the causal sites). For this set of eQTNs we classified each SNP according to its position within the corresponding gene: i.e., the SNP was either in the first, internal or last exon, or it was intronic, or intergenic. We tested each eQTN for association both at the gene level (for the gene identified by the Illumina analysis) and at the exon level (for each exon from that gene that was within 10 kb of the eQTN). Each exon-level test was treated as an independent test.

**Figure 3:**
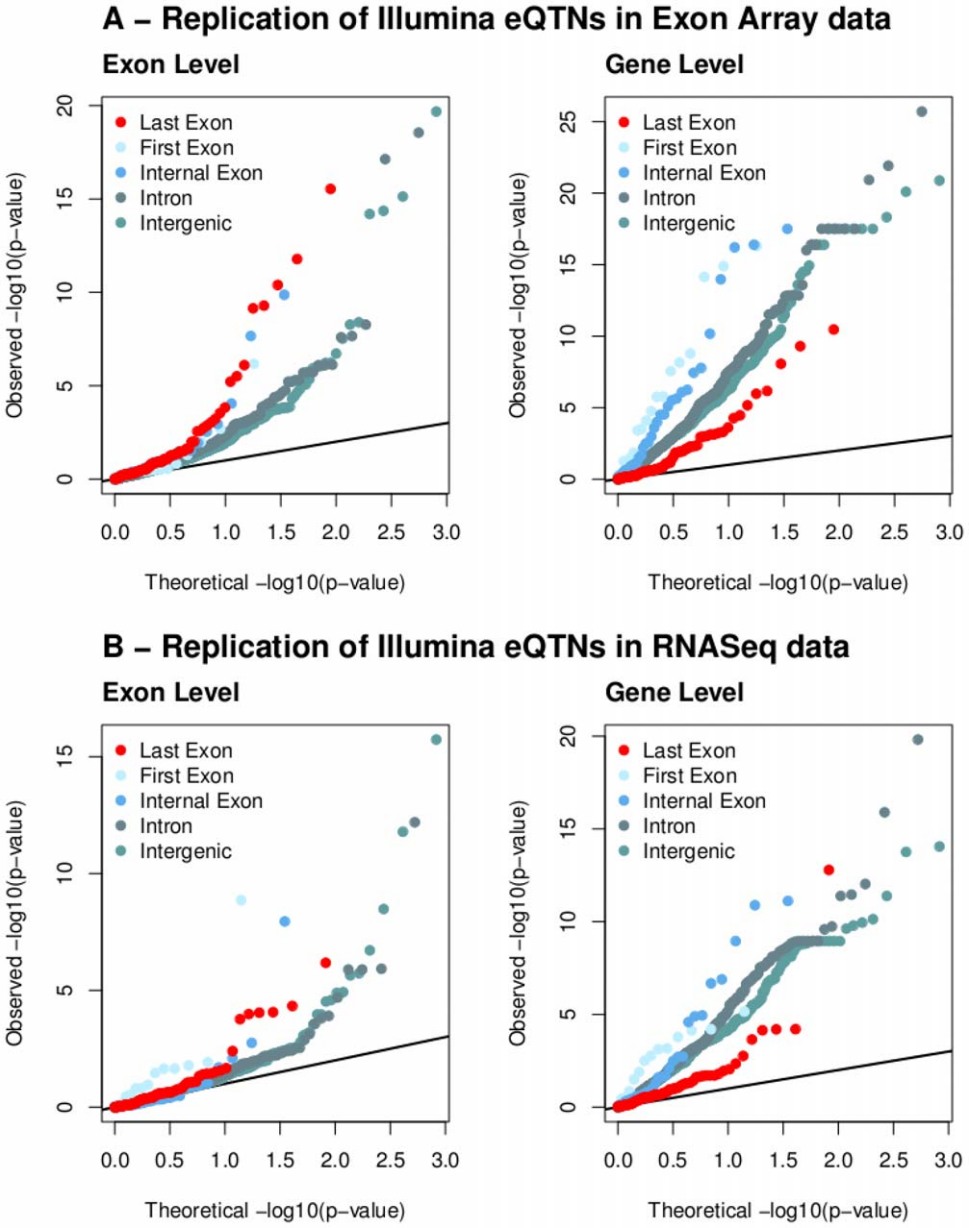
Illumina last exon expression-QTLs are more likely to be splicing-QTLs. We determined the most significant SNP for each Illumina eQTL, and then tested every such SNP for association at the gene- and exon-levels using the Affymetrix and RNA-seq data. Here we show QQ-plots for these Illumina eQTNs in the exon-level analysis (left) and the gene-level analysis (right), using the Affymetrix exon array data (top) and RNA-seq data (bottom). The color codes correspond to 5 exclusive categories of the Illumina eQTNs with respect to the target gene: intragenic, exonic “(first, internal and last) or intronic (intron). Note that last-exon Illumina eQTNs tend to replicate well at the exon level, but poorly at the gene level, suggesting that these are frequently exon-QTLs but infrequently gene-QTLs.

Figure 3A (left panel) shows clearly that last exon Illumina eQTNs are more likely than other Illumina eQTNs to be sQTNs in the Affymetrix data. Conversely, last exon Illumina eQTNs are less likely than other categories of SNPs to replicate at the gene level in the Affymetrix data (Figure 3A, right panel). Although less striking, Figure 3B shows that the RNA-seq data exhibit a very similar pattern. (Note that the RNA-seq analysis of splicing-QTLs is thought to be underpowered in this data set, except for the most highly expressed genes (see Supplementary Figure 15 of [13]), which may explain why the enrichment of last-exon QTLs is lower for the RNA-seq data than for the Affymetrix data.)

**Figure 4:**
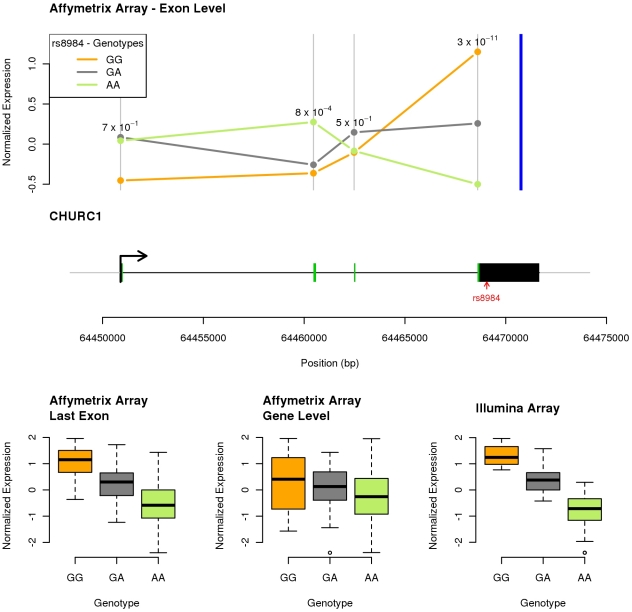
SNP rs8984, located within the last exon of gene CHURC1, primarily affects expression of the last exon, but is interpreted by the Illumina analysis (which has only one probe in this gene) as a gene level QTL. For each panel, we display quantile-normalized expression levels. Data for each genotype at SNP rs8984 are repre- sented with the same color code (orange, grey and green) for all the panels. The top panel plots the mean exon expression levels along the gene as measured by the Affymetrix probes and provides on top of each exon the p-value for the association between the exon expression levels and the SNP genotypes. The blue vertical bar indicates the position of the single Illumina probe. The middle panel is a schematic representation of the gene: exons are plotted as black/green rectangles where the green color indicates coding regions. The position of SNP rs8984 is indicated by a red arrow. The bottom panel provides the box plots corresponding to each analysis: from left to right, specific Affymetrix last exon expression levels (p-value = 3×10^−11^), Affymetrix gene expression levels (p-value = 0.04) and Illumina gene expression levels (p-value = 3×10^−27^).

As an illustration, Figure 4 provides an example of a highly significant Illumina eQTL (p = 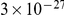), for which the most significant SNP lies within the 3′ UTR. However, analysis of the Affymetrix data indicates that the effect of this SNP is primarily through an exon-specific effect on the last exon (and indeed there may be a separate *opposite* effect on exon 2). In summary, we interpret Figure 3 as evidence that the 3′ peak of eQTLs that was detected in the Illumina data is largely due to a large number of exon-level QTLs that were detected by 3′ UTR probes in the Illumina arrays.

### eQTN enrichment within exons is reproducible between platforms

In our previous work, we also reported a 2-fold enrichment of eQTNs within internal exons compared to introns [9]. Given our newer observations regarding the TES peak, we thus asked whether this exon enrichment is robust across platforms. To evaluate this, we performed a gene-level analysis of all three data sets using our empirical Bayesian framework (see Materials and Methods). We considered four different nested models for the distributions of eQTNs:

1. TSS: the model accounts only for distance from the TSS;
2. TSS, intragenic: same as previously plus an annotation for SNP being within the transcribed region of the target gene;
3. TSS, intron, exon: as previously but the intragenic annotation is split into exclusive intron and exon categories;
4. TSS, intron, exon, last exon: as previously but the exon category is split into exclusive exon (except the last) and last exon categories.

For each of the three data sets, we ran each model separately and then selected the best model based on the Akaike Information Criterion (AIC).

Figure 1D plots for each data set the difference between each model and the best one (i.e., the model with the smallest AIC value). Table 1 also reports the odds ratio estimates of the parameters under each model and for each dataset. As noted above, Model 3 (which includes the special last exon effect) is strongly favored by the Illumina data, weakly favored by the Affymetrix data, and weakly disfavored by the RNA-seq data. However, in contrast, all three data sets agree that Model 1 is significantly better than Model 0 (implying a general enrichment of eQTNs within transcribed regions), and that Model 2 is significantly better than Model 1 (implying an enrichment of eQTNs within exons compared to introns).

**Table 1:** eQTN enrichment within exons is strongly supported by all three datasets while there is relatively weak evidence that the last exon is special.

Model	Annotation	Odds Ratio Estimates [95%CI]		
		Illumina	Affymetrix	RNA-seq
1	intragenic/intergenic	7.51 [6.70, 8.43]	4.25 [3.29, 5.53]	9.12 [6.36, 13.41]
2	exon/intron	12.13 [10.78, 13.60]	11.13 [8.54, 14.33]	6.68 [4.37, 9.89]
3	exon (except last)/intron	5.95 [5.05, 6.96]	8.67 [6.42, 11.49]	7.29 [4.69, 10.94]
3	last exon/intron	28.66 [24.71, 33.13]	21.46 [13.69, 31.94]	4.03 [0.86, 10.50]

The table displays the odds ratio estimates together with their corresponding 95% confidence intervals, as estimated by the empirical Bayesian model (see Methods) for each expression dataset (Illumina, Affymetrix, RNA-seq). Model 1 (TSS, intragenic) estimates the odds ratio that a SNP inside the transcribed region is an eQTN compared to a SNP outside the transcribed region (controlling for distance from TSS). For Model 2 (TSS, intron, exon) and Model 3 (TSS, intron, exon, last exon) we used the intron annotation as the reference: the reported exon and last exon odds ratio can then be interpreted as the relative odds that a SNP within these regions is an eQTN with respect to an intron SNP at the same distance from the TSS.

## Discussion

In this analysis we have shown that the sharp peak of eQTNs previously observed at the TES in Illumina eQTL data [9] appears to be largely driven by SNPs with exon-specific effects. Our results highlight the point that for arrays that probe only a single exon of each gene, it is not possible to distinguish between gene-level and exon-level QTLs. It appears that the majority of eQTLs detected using the Illumina arrays are in fact at the gene-level; however the number of exon-level QTLs is high enough to substantially shift the distribution of QTNs and to complicate the interpretation of eQTLs. In contrast, the gene-level RNA-seq data (and to a lesser extent the Affymetrix data) should be much less sensitive to exon-level effects for most genes, except perhaps when one exon represents a large fraction of the total gene length.

Unlike the TES signal, however, we find that the previously reported enrichment of eQTNs within exons compared to introns [9] is verified in both the Affymetrix and RNA-seq data sets. This suggests that there is an enrichment of regulatory elements within exons. A natural hypothesis is that a fraction of these exonic elements may affect properties of the processed mRNA, rather than affecting transcription rates. These exonic elements may be involved in processes such as promoting mRNA stability or degradation, although further work will be required to test this.

For all three data sets, we found that there is an enrichment of eQTNs within transcribed regions, controlling for distance from the TSS. That is, a SNP at a distance 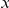 kb downstream from the TSS of gene 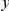 is more likely to be an eQTN if it lies inside the transcribed region of 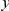 than if it does not. This suggests that long genes may, on average, establish larger domains of cis-regulatory control than do short genes.

In summary, we have shown that the TES peak of eQTLs that was observed previously was most likely driven by QTLs affecting the last exon only. In addition, we have confirmed the enrichment of eQTNs within exons compared to introns; and in transcribed regions compared to downstream intergenic regions, controlling for distance.

## Methods

### Genotype data

For this project we used data from 210 unrelated individuals studied in Phases I and II of the HapMap Project (i.e., all the Chinese and Japanese individuals plus the parents from the Yoruba and CEU trios). The genotype estimates were based on a combination of the 1000 Genomes and HapMap data. These genotypes should include most common variants in the non-repetitive fraction of the genome. For all HapMap SNPs we used the HapMap genotype calls from release 24 of HapMap Phase II [14]. For 141 of these individuals (44 Yoruba (YRI), 30 Japanese (JPT), 29 Han Chinese (CHB) and 43 CEPH (CEU)), we used additional data from the final SNP call set of the 1000 Genomes Consortium pilot data (released March 2010) [15]. Note that for sites that are in both Phase I/II of HapMap and in 1000 Genomes, we used the HapMap data, since these are available for all 210 individuals and are likely to have slightly lower error rates. For the remaining 69 individuals we imputed genotypes at 1000 Genomes SNPs using BIMBAM [16], [17]. We excluded SNPs with MAF 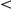
1%. Our final SNP data set consisted of a total of 3.3 M HapMap SNPs and a further 11.3 M 1000 Genomes SNPs.

### Expression data

All the expression datasets were preprocessed using the same gene models, based on the hg18 Ensembl gene annotation track downloaded from the UCSC web site on 12/31/2009.

#### Illumina gene array

We used data from the 210 unrelated individuals in Stranger *et al.* (2007) [6] (i.e., excluding children from the YRI and CEU trios). We first remapped the probes from the array to build 36 (hg18) of the human genome using MAQ [23], selecting only those probes which matched a single unique location with zero mismatches. Of the 47,296 probes on the array, 41,729 fulfilled these criteria. We next selected only those probes that overlapped an annotated exon or exon-exon boundary. We found that 18,478 probes mapped to known exons.

Then we removed “non-expressed” probes by visual inspection of a median versus median absolute deviation (MAD) scatter plot augmented with the fraction of expressed genes at a given MAD value as derived from the RNA-seq datasets. From this visualization it is clear that there are two populations of probes, one “expressed” population with moderate to high MAD values and a “non-expressed” population of probes with low MAD values. This analysis indicated that probes with low MAD are generally non-expressed. We thus removed 8,214 “non-expressed” probes from the original set which yields a core set of 10,264 probes.

Since expression measurements are susceptible to large technical variability and since we are looking only at cis-eQTLs, we performed a principal component-based adjustment of the expression dataset similar to what it has been previously described by our group [13] and in other studies [24]–[27]. To do so we first adjusted each expression measurement 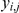 by subtracting the population mean value for gene 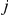 in the population of individual 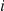, in order to bring all populations to the same mean value. We next performed principal components analysis on the expression matrix. By doing 100 permutations of the population-adjusted probe expression values we derived an empirical distribution of the PC eigenvalues under the null hypothesis assuming independence of all probes. We then selected the optimal number of PCs by choosing the last PC for which the observed eigenvalue exceeds the upper 97.5% percentile of the empirical null distribution as derived by our bootstrap procedure. With this procedure we retained 26 PCs that we then regressed out probe-by-probe using an elastic-net [28] linear model which also includes the population and the sex covariates for each sample. Briefly, an elastic-net regression was performed for each probe by using an in-house C implementation of the standard LARS-EN procedure [28]. Regarding the tuning parameter 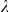 we used a discrete grid of 6 points 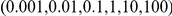 corresponding to almost Lasso behavior (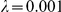) to almost ridge behaviour (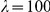). For each probe, the optimal value of 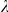 was obtained by applying a leave-one out cross-validation procedure: for each individual left out we thus computed the squared difference between the actual probe expression value of this individual and the one predicted by the elastic-net linear model learned only with the other individuals. The optimal value of 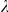 is then the one that minimizes the residual sum of squares across individuals. We then derived for each probe the corresponding corrected expression levels by computing the residuals from the optimal elastic-net linear regression model. These corrected probe expression levels were then used for all subsequent analyses. Finally, we removed all probes that overlapped with any known variant including SNPs, copy number variants and short insertions/deletions [15], [20].

#### Affymetrix exon array

We downloaded the raw Affymetrix Human Exon 1.0 ST array CEL files published by Huang *et al.* (2007) [18] from GEO (GSE7792). As for the Illumina array, we first remapped the probes to build 36 (hg18) of the human genome using MAQ [23], selecting only those probes which matched a single unique location with zero mismatches. The probe sequences were obtained from the Affymetrix website (http://www.affymetrix.com/Auth/analysis/downloads/na25/wtexon/HuEx-1_0-st-v2.probe.tab.zip). Of the 5,431,924 probes on the array, 4,839,062 fulfilled these criteria. We next selected only those probes that overlapped an annotated exon while removing probes interrogating exons shared by at least two distinct genes (66,347 probes). This yields a core set of 1,355,061 single gene exonic probes.

We performed GC-bin background correction of each array followed by a quantile normalization on natural scale within each population (CEU and YRI) using an in-house implementation. We then removed “non-expressed” probes defined as probes with a median normalized intensity level below 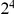 within both populations (this is based on visual inspection of both the distribution of the normalized median probe expression levels within each population and the probability that the probe intensity is not drawn from the distribution of background intensities empirically defined by the corresponding matched GC content negative-controls probes). For the final set of 1,060,605 pre-processed exonic probe expression levels and as for the Illumina dataset, we performed a principal component-based adjustment by combining the expression levels from both populations. Using the same approach as previously described we thus regressed out the effect of 35 PCs including population and sex covariates within the elastic-net model. Finally, as for the Illumina dataset, we used a conservative approach and removed all the probes which overlap with a 1000 Genomes SNP inside yielding to a final core set of 444,306 exonic probes.

To compute entire-gene expression levels we used a simple summarization approach based on the median polish procedure [29], [30]. Briefly we considered all the probes that span the entire transcribed region and build a two-way layout matrix where the probes are in columns and the samples in rows. We then fit the additive model 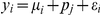, where 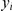 is the gene expression level for sample 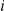, 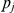 is the effect of probe 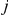 and 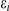 is the residual, by iteratively removing the median of rows and columbs until convergence. As mentioned in the main text we also considered an alternative by applying the median polish procedure first at the exon level followed by a second median polish procedure on the sample x exon expression matrix to get the gene expression levels. Finally, to perform our splicing-QTN mapping we computed splicing exon expression levels as described in [31]: specific exon expression levels are thus derived from the residuals of the gene level median polish procedure. Note that for sQTN mapping we used only exons from genes having at least two exons with expression level measurements (85,524 exons from 6,884 distinct genes).

#### RNA-seq analysis

We obtained published RNA-seq data from 60 CEU individuals and 75 YRI individuals [13], [19]. Both data sets were pre-processed roughly as described in the Supplementary Information of Pickrell et al. [13]. We performed the correction for GC content as described using all 135 individuals together.

From the original 241,639 exons we removed 118,548 exons for which the median counts within both populations were 0. Using this core set of 123,091 exon level expression measurements we performed a PCA-based adjustment but this time separately within each population (since sequencing was performed in two distinct environments each one may have its own hidden factor structure). Thus, including sex as a covariate, we regressed out 12 specific PCs for the YRI dataset and 16 other specific PCs for the CEU dataset.

For subsequent analyses, we used only the 43 CEU and 59 YRI samples that were included within our genotype dataset. As for the expression arrays, gene expression levels were computed by applying a median polish procedure onto the sample x exon expression level matrix, thus removing exon specific effects. Similarly specific exon expression levels have thus been derived from the residuals of the gene level median polish procedure [31]. As for the Affymetrix dataset, sQTN mapping is based only on exons from genes having at least two exons with expression level measurements (91,960 exons from 8,658 distinct genes).

### Analyses

For the technical details of the statistical analyses we invite the reader to refer to our previous article [9]. The main differences here are:

- for eQTN mapping we restricted the cis-candidate region to 100 kb around both gene ends (instead of 500 kb),
- for sQTN mapping we used a 10 kb window around both exon ends, since it has been previously show that sQTNs are mainly concentrated nearby the spliced-exon [13].

The exon-level FDR for the sQTN analysis has been obtained in the same way as the gene-level FDR described in [9]. Finally, the extension of our empirical Bayesian model is detailed in [11].

## Supporting Information

Table S1
**Summary of the three expression datasets used in this study.** For Illumina and Affymetrix datasets the number of probes corresponds to the final number of probes used after filtering. Both the number of eQTNs and sQTNs are reported for an empirically estimated FDR of 5% (see Material and Methods). For eQTNs, the corresponding p-value cutoffs are: i) Illumina (p-value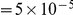), ii) Affymetrix (p-value 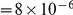), iii) RNA-seq (p-value 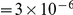). For sQTNs the p-value cutoffs are: i) Affymetrix (p-value 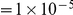), ii) RNA-seq (p-value 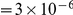). The smaller number of sQTLs in the RNA-seq data may be due to lower power in the RNA-seq data, except for the highest expressed genes (Supplemental Figure 15 of [13]).(PDF)
