# Supplementary material for: Exon-Specific QTLs Skew the Inferred Distribution of Expression QTLs Detected Using Gene Expression Array Data
Source: PLoS One. 2012 Feb 16;7(2):e30629. doi: 10.1371/journal.pone.0030629 (PMC3281037; doi:10.1371/journal.pone.0030629)
Supplement: Table S1 — Summary of the three expression datasets used in this study. For Illumina and Affymetrix datasets the number of probes corresponds to the final number of probes used after filtering. Both the number of eQTNs and sQTNs are reported for an empirically estimated FDR of 5% (see Material and Methods). For eQTNs, the corresponding p-value cutoffs are: i) Illumina (p-value), ii) Affymetrix (p-value ), iii) RNA-seq (p-value ). For sQTNs the p-value cutoffs are: i) Affymetrix (p-value ), ii) RNA-seq (p-value ). The smaller number of sQTLs in the RNA-seq data may be due to lower power in the RNA-seq data, except for the highest expressed genes (Supplemental Figure 15 of [13]). (PDF) [file pone.0030629.s001.pdf]

Expression Dataset	References	Samples	Probes	Exons	Genes	eQTNs	sQTNs
Illumina Gene Array	[?]	CEU(60), CHB(45), JPT(45), YRI(60)	8,613	8,613	7,848	2,896 (37%)	NA
Affymetrix Exon Array	[?]	CEU(58), YRI(59)	444,306	90,245	7,470	524 (7%)	645 (0.75%)
RNA-seq	[?, ?]	CEU(43), YRI(59)	NA	103,952	9,201	214 (2%)	142 (0.15%)

Table S 1. Summary of the three expression datasets used in this study. For Illumina and Affymetrix datasets the number of probes corresponds to the final number of probes used after filtering. Both the number of eQTNs and sQTNs are reported for an empirically estimated FDR of 5% (see Material and Methods). For eQTNs, the corresponding p-value cutoffs are: i) Illumina (p-value= 5×10^{-5}), ii) Affymetrix (p-value = 8×10^{-6}), iii) RNA-seq (p-value = 3×10^{-6}). For sQTNs the p-value cutoffs are: i) Affymetrix (p-value = 1×10^{-5}), ii) RNA-seq (p-value = 3×10^{-6}). The smaller number of sQTLs in the RNA-seq data may be due to lower power in the RNA-seq data, except for the highest expressed genes (Supplemental Figure 15 of [?]).
